# A mixed-methods pilot study exploring midwives’ job satisfaction: Is being of service to women the key?

**DOI:** 10.18332/ejm/146087

**Published:** 2022-04-19

**Authors:** Kim Oliver, Sadie Geraghty

**Affiliations:** 1 School of Nursing, Midwifery, Health Sciences and Physiotherapy, University of Notre Dame Australia, Fremantle, Australia

**Keywords:** midwives, job satisfaction, workforce, shift-work

## Abstract

**INTRODUCTION:**

The purpose of this research project was to investigate midwives’ job satisfaction in Australian maternity care settings.

**METHODS:**

A mixed methods pilot study using the convergent parallel design, and a mixed-methods approach was used for this study. The Nursing Workplace Satisfaction questionnaire was used to collect data online via social media platforms, and consisted of Likert Scale responses, and both closed and opened ended questions.

**RESULTS:**

The quantitative results noted an overall positive result to participants’ job satisfaction, however there were areas that participants reported as problematic. These areas were delved into further via the results of the qualitative data which highlighted eight themes that explored the participants’ perception of the worst things that impacted upon their job satisfaction, and also the best things which impacted in relation to their current jobs.

**CONCLUSIONS:**

This study revealed factors including staff shortages, being time-poor, missing basic human rights like meals and comfort breaks which were linked to midwives’ dissatisfaction with their jobs in Australia. The study also identified that midwives valued being of service to women, and that this factor was a driving force in job satisfaction.

## INTRODUCTION

Providing quality, safe maternity care is dependent upon the provision of a highly skilled midwifery workforce^1,2^. However, healthcare organizations are struggling to retain midwives, which is resulting in global workforce shortages^3-5^. According to The State of the World’s Midwifery Report (2021) these shortages are expected to worsen over the next few years following the global COVID-19 pandemic^6^ due to burn-out and loss of life^7,8^, and as the midwifery workforce ages^9^. The average age of a midwife in Australia is 48.8 years, with 55.6% of the working population being over 50 years of age^10^. Despite efforts to improve the midwifery shortage in Australia, the problem still exists and is worsening^5^. It is therefore imperative that healthcare agencies explore midwives’ job satisfaction as it has been associated with staying in the midwifery profession^11^, and has an impact upon cost effectiveness to organizational, neonatal and maternal health outcomes^12^.

Operationalizing the concepts and assessing the job satisfaction of midwives is a complex issue, as this can relate to numerous variables including working hours, leadership style, organizational structure, women and neonatal outcomes, or it may be limited to the specific function of a job or personal characteristics^13,14^. It appears an individual’s perception of job satisfaction has changed over time, along with varying definitions, e.g. job satisfaction now includes factors such as work environment, organizational demands, and professionalism, with the personal job satisfaction of midwives included as one of the constructs^15,16^. Some argue this lack of clarity is due to job satisfaction being a multi-faceted entity, including factors such as work environment and leadership styles, rather than a singular element^17,18^.

Maslow^19^ viewed job satisfaction as a human need, which was fulfilled by the individual’s occupation, whilst others perceive it as everything that is good about their job. Castaneda and Scanlan^20^ concluded that job satisfaction relates to three specific areas, firstly, autonomy (scope of practice, teamwork, management and co-worker support, trust between staff, and education). Secondly, providing care (relationships with women/clients, acknowledgement of care from women/clients and family members, and the perception of providing good care), and interpersonal relationships (relationships between doctors, nurses and other health professionals)^20^. Liu et al.^21^ state that job satisfaction relates to the ‘happiness, or enjoyment that an employee feels when doing his or her work’, which encapsulates the areas identified by Castaneda and Scanlan^20^. Recent studies have been conducted to address the gaps in research exploring the relationship between midwives’ job satisfaction^17^, quality of work life^15^ measurements of wellbeing of midwives and reasons to stay in midwifery^11,12,22^. Results published from these studies reveal that there is a positive and significant correlation between the intent to stay or not in the profession and job satisfaction.

From a slightly different perspective, researchers need to be clear of the variables they wish to explore and ensure that they are using the correct tool prior to commencing their research studies due to the blurring of inclusivity of this phenomenon^23^. Over time, job satisfaction tools appear to have changed focus, to include work environment, organizational demands, and professionalism, rather than the personal job satisfaction of the midwife. Some argue this is due to job satisfaction evolving and now including different aspects of the work environment, and also encompassing leadership styles^24^, rather than a single entity. Previous studies have identified different factors that may also contribute to job satisfaction, such as positive self-appraisal^14^. Thus, whilst many factors appear to contribute to extrinsic job satisfaction, intrinsic satisfaction seems to rely on self-reflection, positive self-appraisal, self-recognition and a sense of competency^14^. Moreover, if the correct tools are not used, then findings will undoubtedly be flawed, and this important aspect of a midwife’s work will be underestimated or underreported.

This pilot study aimed to investigate midwives’ job satisfaction in maternity care settings by posing the question ‘what does job satisfaction consist of for midwives in Australian maternity care settings?’ and using the previously validated Nursing Workplace Satisfaction questionnaire as the tool to collect data for the study.

## METHODS

A mixed-methods pilot study using the convergent parallel design, and a mixed-methods approach was used. The quantitative and the qualitative components were conducted sequentially. Both the quantitative and qualitative data were utilized to enhance the description and understanding of midwives’ job satisfaction in maternity care settings. The Nursing Workplace Satisfaction questionnaire and two open-ended qualitative research questions were used to collect data in this study: ‘Can you tell us about the best things about your job?’ and ‘Can you tell us about the worst things about your job?’.

### Methodology

The Nursing Workplace Satisfaction questionnaire was reproduced with kind permission from Greg Fairbrother^25^. The study was conducted online through the Qualtrics™ online platform. Social media platforms such as Twitter and Facebook were used to recruit participants. These were closed group midwifery forums, and participants were asked to share the link to the study with their own midwifery networks. Questionnaire completion time was estimated to be approximately twenty minutes, although it was not mandatory for participants to answer all questions; an in-depth information sheet was provided to help motivate participants.

### Participants

The unit of analysis for this study were registered midwives working in maternity care settings in Australia. Forty-four respondents completed the anonymous questionnaire via an online platform. As this study was a pilot study, a statistical formula was used to determine the sample size required^26^. Viechtbauer et al.^26^ suggest that if a problem exists, using a 5% probability will detect its existence, and using a confidence level of 95% will identify the problem. This study used a 5% probability and a confidence level of 89% to determine that 43 participants was adequate for this pilot study. As Sauro and Lewis^27^ note, using a confidence level greater than 80% is sufficient when the aim is to obtain general feelings from a group of participants.

Permission to undertake the study was obtained from the University Human Research Ethics Committee. An information sheet was available to participants, outlining the purpose of the study. The completed questionnaires were anonymous and unable to be identified. A formal consent form was signed by each participant before commencement of the questionnaire. On completion of the study, the data were stored securely as per the university’s policy for research data management.

### Questionnaire

The Nursing Workplace Satisfaction questionnaire included 18 statements using a 5-point Likert scale with choices ranging from strongly agree to strongly disagree. Open-text qualitative questions provided respondents the opportunity to comment on what was the best components of their job and what were the worst. It consists of intrinsic questions which focused on how the individual enjoyed their job, if it gave them satisfaction and meaning. It sought to explore if participants thought their job gave them an opportunity to show what they were worth, and if they were enthusiastic about their present job. The questionnaire also explored extrinsic factors such as did they perceive they had enough time to deliver good patient care to women, enough support from colleagues, and the busyness of the environment or feelings of isolation or lack of confidence as a clinician, and the final domain focused on the relational aspects of job satisfaction which include the perception of making friends amongst their colleagues, and feeling like they belong, and liking the people they work with.

The tool has been validated in the nursing profession^25^ and found suitable for use in a midwifery context.

### Data collection

Participants had six weeks to complete the online survey. Participation was voluntary and participants were made aware, via the provided information, that they had the right to withdraw at any time without prejudice or need for justification. As the surveys were anonymous, any information submitted was not able to be returned to the participants as the collected data were de-identifiable.

### Statistical analysis

Data were collected online via social media. To ensure that an in-depth understanding occurred, a convergent parallel approach was taken as part of the mixed-methods design. This approach necessitates that the quantitative and qualitative variables denoted as QUAL + QUAN^28^, occur at the same time within the research process, and weigh equally within interpretation of the collective results^29^. In line with this approach, the quantitative data were analyzed via the Qualtrics^TM^ platform, and the qualitative data were analyzed using content analysis. Qualitative content analysis was organized by the researchers examining the data for patterns of themes from the thoughts and perceptions of the respondents^30^. There were two open-ended questions with free text boxes for midwives to provide their own experiences/perceptions when answering. After analysis of the quantitative data which fell into three domains, content analysis of the qualitative data identified five subthemes relating to the worst things that impacted upon job satisfaction, and three subthemes describing the best thing impacting upon job satisfaction.

## RESULTS

Forty-four participants completed either all or parts of the survey. The survey focused on three domains: Intrinsic, Extrinsic and Relational (Table 1).

**Table 1:** Participants’ responses to the Nursing Workplace Satisfaction Questionnaire

*Types of Questions*	*Question*	*Response rate n*	*Fully agreed n (%)*	*Agreed n (%)*	*Partly agreed n (%)*	*Disagreed n (%)*	*Definitely disagree n (%)*	*Mean*	*SD*	*Variance*
**Intrinsic**	Q1. My job gives me a lot of satisfaction	35	3 (8.57)	6 (45.71)	14 (40)	2 (5.71)	0 (0)	2.43	0.73	0.53
	Q2. My job is very meaningful for me	37	18 (48.65)	16 (43.24)	3 (8.11)	0 (0)	0 (0)	1.59	0.63	0.40
	Q3. I am enthusiastic about my work	35	2 (5.71)	11 (31.43)	13 (37.14)	9 (25.71)	0 (0)	2.83	0.88	0.77
	Q4. My work gives me the opportunity to show	34	1 (2.94)	8 (23.53)	12 (35.29)	11 (32.35)	2 (5.88)	3.15	0.94	0.89
	Q5. In the last year, my work has grown more interesting	35	2 (5.71)	5 (14.29)	6 (17.14)	19 (54.29)	3 (8.57)	3.46	1.02	1.05
	Q6. It is worthwhile to make an effort in my job	36	11 (30.56)	12 (33.33)	6 (16.67)	7 (19.44)	0 (0)	2.25	1.09	1.19
**Extrinsic**	Q7. I have enough time to deliver good care to patients	35	0 (0)	1 (2.86)	6 (17.14)	12 (34.29)	16 (45.71)	4.23	0.83	0.69
	Q8. I have enough opportunity to discuss patient problems with my colleagues	34	0 (0)	2 (5.88)	7 (20.59)	17 (50.00)	8 (23.53)	3.91	0.82	0.67
	Q9. I have enough support from colleagues	32	1 (3.13)	7 (21.88)	9 (28.13)	9 (28.13)	6 (18.75)	3.38	1.11	1.23
	Q11. I feel able to learn on the job	36	1 (2.78)	14 (38.89)	12 (33.33)	6 (16.67)	3 (8.33)	2.89	0.99	0.99
	Q12. I feel isolated from my colleagues at work	33	3 (9.09)	7 (21.21)	10 (30.30)	10 (30.30)	3 (9.09)	3.09	1.11	1.23
**Relational**	Q15. It’s possible for me to make good friends among my colleagues	35	8 (22.86)	12 (34.29)	12 (34.29)	2 (5.71)	1 (2.86)	2.31	0.98	0.96
	Q16. I like my colleagues	34	8 (23.53)	14 (41.18)	12 (35.29)	0 (0)	0 (0)	2.12	0.76	0.57
	Q17. I feel that I belong to a team	36	5 (13.89)	14 (38.89)	11 (30.56)	6 (16.67)	0 (0)	2.50	0.93	0.86
	Q18. I feel that my colleagues like me	36	6 (16.67)	20 (55.56)	10 (27.78)	0 (0)	0 (0)	2.11	0.66	0.43
**Stand-alone**	Q10. I would function better if it was less busy on the ward/unit	38	28 (73.68)	7 (18.42)	3 (7.89)	0 (0)	0 (0)	1.34	0.62	0.38
	Q13. I feel clinically confident	35	11 (31.43)	13 (37.14)	9 (25.71)	2 (5.71)	0 (0)	2.06	0.89	0.80
	Q14. I like the way my ward is run	32	0 (0)	0 (0)	9 (28.13)	14 (43.75)	9 (28.13)	4.00	0.75	0.56

The intrinsic domain consisted of questions 1-6; extrinsic domains were captured by questions 7, 8, 9, 11, 12, and finally the relational domain was captured by questions 15– 18 of the Nursing Workplace Satisfaction questionnaire^25^.

Questions 10, 13 and 14 were removed as Fairbrother et al.^25^ state that question 10 is addressed by question 7 as both capture workplace feelings, and questions 13 and 14 form stand-alone questions which do not fall into an extrinsic domain.

Overall, the intrinsic findings were positive towards how much the participants enjoyed their job. However, when asked the question: ‘in the last year, my work has grown more interesting’ (question 5), 19 participants disagreed (n=35). The least favorable was the extrinsic domain which relates to doing your job and especially question 8: ‘I have enough opportunity to discuss patient problems with colleagues’ with 17 participants (n=34) disagreeing.

The relational domain relates to the people the participants work with, and was viewed as favorably by participants, with 20 responses (n=36) agreeing with question 18: ‘I feel that my colleagues like me’.

Question 13: ‘I feel confident as a clinician’ highlighted those participants that felt confident as clinicians, with 24 respondents (n=35) reporting either fully agree or agree to this question.

Finally, question 14: ‘I like the way my ward is run’ was highlighted by 32 participants who answered the question; 9 participants partly agreed with this view, 14 disagreed and 9 definitely disagreed.

Fairbrother et al.^25^ suggest that question 10 be removed at the results stage, as it is viewed that this information is captured by question 7. Twenty-eight participants (80%) disagreed or definitely disagreed with the question that they ‘have enough time to deliver good care to patients’, which compares favorably with the findings of question 10 which seeks to find out if participants would ‘function better if it was less busy on the ward’, to which 35 (92%) participants either fully agreed or agreed, which therefore validates Fairbrother et al.^25^.

The qualitative results revealed five subthemes describing the worst things that impacted upon job satisfaction, and three subthemes describing the best thing impacting upon job satisfaction in the participants’ current jobs.

### Theme 1: Worst things impacting upon job satisfaction

*Subtheme 1: ‘We are desperately short of staff.’*

The midwifery respondents described a shortage of staff was one of the worst things impacting upon job satisfaction. They identified that ‘staff ratios’, ‘a lack of staff’ and ‘being constantly understaffed’ were having serious consequences in their roles, with one midwife stating: ‘it is now dangerous on some of the wards’ (AS21).

‘Poor retention of staff’ was also highlighted, as were ‘being on call’ and ‘being on call 24 hours’, which led to ‘not being able to switch off, causing burn-out’.

*Subtheme 2: ‘Shift work isn’t working for many of us.’*

Respondents identified that shift work contributed to dissatisfaction with midwifery jobs, describing: ‘inflexible work hours’ being ‘not life-style friendly for midwives or women’ (AS12), and that rosters and long shifts did not fit in with young families.

*Subtheme 3: ‘Management don’t care.’*

The impact of management was identified as a factor in midwives’ job satisfaction, with ‘micro-managing’ and ‘lack of support from management’ being a common response. Statements of ‘management doesn't care’, ‘bad management’ and ‘a lack of respect from the hospital management and managers’ (AS6), were also identified. Many respondents also stated that ‘management make decisions without consulting the staff’(AS27).

*Subtheme 4: ‘We have no time.’*

Time-related issues attracted the most comments from the midwifery respondents and appeared to be a major factor in job dissatisfaction. ‘Lack of time to do the job properly’, an ‘increase in women/patient numbers’ and ‘not having enough time to make a difference to women’ were some of the most common responses; ‘babies not in patient ratios’, ‘pushing on the clock unnecessary interventions’ and ‘not giving care you should give’ were also cited. Many respondents said they were ‘time poor’ and complained about the ‘lack of time spent with women’. Two respondents said they felt ‘unsafe’ because they were ‘overworked’ on many shifts, and that they were ‘not able to provide the best evidence-based care due to time constraints’ (AS6), and ‘unable to provide the necessary support to women because of being time poor’ (AS23).

Many respondents stated that ‘I don't have enough time on the wards to give women the time they deserve’ (AS9) and ‘not being able to give women continuity and time they should have due to staffing/care model constraints’ (AS32).

*Subtheme 5: ‘We are missing our basic rights.’*

Most respondents described ‘not getting breaks’, and ‘missing meals’ whilst on shift. This was seen as an important issue in being dissatisfied with their current jobs. The midwives stated a ‘lack of relief for unexpected leave’ or ‘long periods of planned leave’ left them feeling ‘physically exhausted’. One respondent described ‘feeling constantly blamed and feeling gutted that we constantly have to beg and justify basic rights such as lunch breaks and toilet breaks. I'm exhausted picking up other's work’ (AS 31).

### Theme 2: Best things impacting upon job satisfaction

*Subtheme 1: ‘Midwifery models of working.’*

The midwifery respondents described working within midwifery models of care, increased their satisfaction in their current jobs. Many respondents identified ‘continuity of care and the variety of the work’, ‘positive outcomes with minimal interventions’, ‘the teamwork on the ward’, ‘working with women’, ‘working in a woman-centered care way’, and ‘working in the continuity model’ as some of the best things about their roles. Every participant responded with at least one of the responses recorded.

*Subtheme 2: ‘Providing midwifery care is our priority.’*

Midwifery respondents were overwhelmingly clear in that prioritizing midwifery care had a positive impact upon job satisfaction. The participants made a series of similar comments about how providing care was a rewarding aspect of their role; this included ‘working with the women to provide quality care’, ‘the families I care for each day make my job worthwhile’ (AS19), ‘I am passionate about being a midwife and providing quality midwifery care’ (AS7) and ‘I enjoy caring for new mothers’.

*Subtheme 3: ‘Being of service to women.’*

This theme that emerged from the collected data, contained the largest volume of comments from participants. Midwives were unanimous that being of service to the women they provided care to, was the best indicator of their job satisfaction. Every respondent described aspects of the role that made their job enjoyable, with one stating that

‘building relationships with women and their support persons and assisting them to achieve as close to their birthing goals as possible makes me stay in my job’ (AS12),

‘being able to make the most vulnerable time of someone's life a fulfilling and empowering time’ (AS34),

‘the precious moments where I can give good advice, empower a woman to make her own informed choices is so rewarding’ (AS14),

and ‘building rapport with women and making their pregnancy/birth/postnatal experience better’ (AS5).

Many respondents identified ‘helping a woman to have the birth she wanted’ (AS17), ‘advocating for the woman’, and ‘making a difference to women and their families’ as important factors of satisfaction in their jobs.

## DISCUSSION

The results from this study revealed that the overarching concept of providing care, building relationships, advocating for women, helping women to achieve the birth they desired, and making a difference for women, equated to being of service to women, which ultimately appeared to be the main factor for job satisfaction for midwives. Intrinsic factors such as self-reflection, positive self-appraisal, self-recognition, and a sense of competency^14,25^, are noted as being positive within this study. Moreover, intrinsic factors which encompass self-worth, and a sense of achievement are vitally important not only to explore job satisfaction but are predictors of an individual’s job performance. Participants within the current study reported that their job gave them job satisfaction, which is a positive finding, as it is well documented that perceptions of poor job satisfaction increase the likelihood of burnout amongst staff^31,32^. Self-worth is another intrinsic factor that is explored within this study, whereby participants were asked if they felt their ‘job gave them opportunities to show what they were worth’. Interestingly, findings were again positive, which is important, as Maslach and Leiter^33^ identify that when there is any form of mismatch relating to the main areas of a person’s work, such as their perceived control over their job, or performance, and if the warning signs are not recognized or addressed in a timely manner, burnout can ultimately occur.

Extrinsic factors such as support from colleagues, workloads, or training, are all aspects that can impact on job satisfaction^25^. Within the current study, the extrinsic domain has been negatively reported, for example, when asked if participants were ‘given enough opportunity to discuss patient problems’. The midwifery profession focuses on providing women-centered care, having a women focused approach is fundamental to all that midwives do. Frawley et al.^34^ have also reported negative findings relating to extrinsic domains, suggesting that midwives perceived they were time-poor, and this resulted in them not having time to do things well, or adequately address parents’ concerns.

The relational domain explores the dynamics of the team with whom participants work, their relationships and teamwork, and wider network. Having effective social interaction and relationships are crucial to sustaining a motivational momentum and engagement^35^. Overwhelmingly, participants of the current study (100%) agreed with this view, strengthening the findings of Thapa et al.^36^ in a study where they explored the health of nurses and midwives in the workplace, whereby collegial support and teamwork were viewed by participants as inspiring, and crucial, not only to their own health, but for job satisfaction. This substantiates the view of the need, and necessity to nurture and grow an effective workplace relationship and culture^35^.

Eight determinants relating to job satisfaction for midwives were revealed in the qualitative section of this study. The everyday issues impacting upon job satisfaction appeared to stem from a shortage of staff, inflexible shifts, a lack of support from management that caused midwives to be time-poor, unable to have the time to provide the care that women required and that the midwives’ basic needs regarding meal/comfort breaks were being ignored. In the United Kingdom (UK), a campaign by the Royal College of Midwives called ‘Caring For You’ in 2016, aimed to improve the health and safety of midwives, which included the importance of taking meal/comfort breaks^37^. There are not many jobs that do not recognize the importance of being able to take breaks, instead of working continually to the end of a shift. In recent studies, midwives consistently did not have the ability to take meal breaks due to staff shortages^38^, and nurses’ and midwives’ experiences of urinary symptoms at work were found to primarily relate to delayed voiding^39^. These studies, and the results from this study, suggest that the missing of meals and comfort breaks are directly related to a workplace culture that puts women or patients first, and deters self-care of those providing care to others.

The current global staff shortage in all areas of clinical midwifery practice^40^ is affecting the job satisfaction of midwives. In the UK, a recent study suggested that there was a shortage of 3500 full time midwives, and skills, experience and confidence were being lost due to the aging midwifery workforce retiring^41^. In Australia, a recent study identified that the midwifery workforce was impacted by shortages and attrition, which had bearing upon the ability to be a midwife and also negatively affected workplace culture^42^. It has been predicted that the world is facing a shortage of about 0.9 million midwives, according to the latest State of the World’s Midwifery (SoWMy) report, which was released on the International Day of the Midwife in 2021^43^. Therefore, the issue of continuing staff shortages has been shown to negatively impact on midwives’ daily working experience^44^, forcing midwives to provide quick, basic care, rather than focusing on quality midwifery care that forms job satisfaction for most midwives.

Being of service to women, in providing woman-centered care and having time to support women, appears to withstand the impact of other factors that have bearing upon midwives’ satisfaction with their jobs. By being of service to women, midwives are choosing to engage without expectation or reciprocation, which is linked to the midwifery fundamental belief of being ‘with woman’. As a profession, midwifery has a deep-seated service to women and their families which becomes embedded within midwifery curricula via continuity of care experiences and the foundational belief of woman-centered care. Therefore, this core concept that is conceived in midwifery education and training, remains as a major incentive within midwifery job satisfaction.

### Limitations

This study was conducted within Australia and therefore, may not be generalizable to midwives working in other countries. The results also need to be interpreted with caution in regard to other global settings, given the study was conducted solely in Australia, and so may not necessarily be transferrable. As there were only 44 respondents in this pilot study, job satisfaction attitudes may not be generalizable for the Australian midwifery population. This study was undertaken in 2021 during the COVID-19 pandemic, where lockdowns, illness, and the changes to the provision of maternity care may have impacted upon midwives’ time to participate in the study and their attitudes to job satisfaction. We intend to undertake a larger study including international midwives, hoping to explore whether the current issues for midwives are the same as those reported here. The planned study will enable both the context of the COVID-19 pandemic and the views of midwives from multiple organizations to be explored.

## CONCLUSIONS

Exploring midwives’ job satisfaction is an important area of study due to the predicted global shortfall of midwives in the midwifery workforce. This study revealed factors both within the qualitative and quantitative data which linked midwives’ satisfaction with their jobs in Australia, and identified that midwives valued being of service to women, which became evident within the intrinsic factors analysis of the questionnaire, and within the open-ended long answer questions, and, furthermore, this was a driving factor in their job satisfaction. With the looming global shortage of midwives, the satisfaction of midwives in the workforce needs to be a priority to avoid a future crisis in the midwifery workforce.

## Data Availability

The data supporting this research are available from the authors on reasonable request.
